# Quantum paraelectric varactors for radiofrequency measurements at millikelvin temperatures

**DOI:** 10.1038/s41928-024-01214-z

**Published:** 2024-08-05

**Authors:** P. Apostolidis, B. J. Villis, J. F. Chittock-Wood, J. M. Powell, A. Baumgartner, V. Vesterinen, S. Simbierowicz, J. Hassel, M. R. Buitelaar

**Affiliations:** 1 London Centre for Nanotechnology, University College London, London, UK; 2 Department of Physics and Astronomy, University College London, London, UK; 3 Department of Physics, University of Basel, Basel, Switzerland; 4 QTF Centre of Excellence, VTT Technical Research Centre of Finland Ltd, Espoo, Finland

**Keywords:** Electronic devices, Quantum information, Electrical and electronic engineering

## Abstract

Radiofrequency reflectometry can provide fast and sensitive electrical read-out of charge and spin qubits in quantum dot devices coupled to resonant circuits. In situ frequency tuning and impedance matching of the resonator circuit using voltage-tunable capacitors (varactors) is needed to optimize read-out sensitivity, but the performance of conventional semiconductor- and ferroelectric-based varactors degrades substantially in the millikelvin temperature range relevant for solid-state quantum devices. Here we show that strontium titanate and potassium tantalate, materials which can exhibit quantum paraelectric behaviour with large field-tunable permittivity at low temperatures, can be used to make varactors with perfect impedance matching and resonator frequency tuning at 6 mK. We characterize the varactors at 6 mK in terms of their capacitance tunability, dissipative losses and magnetic field insensitivity. We use the quantum paraelectric varactors to optimize the radiofrequency read-out of carbon nanotube quantum dot devices, achieving a charge sensitivity of 4.8 μ*e* Hz^−1/2^ and a capacitance sensitivity of 0.04 aF Hz^−1/2^.

## Abstract (web summary)

Using materials that show quantum paraelectricity, a phenomenon in which ferroelectric order is suppressed at very low temperature, voltage-tunable capacitors can be created for use in sensitive read-out circuits to measure cryogenic quantum devices.

## Main

Radiofrequency (RF) reflectometry is a measurement technique that allows fast and sensitive read-out of charge detectors such as single-electron transistors^1–3^, quantum point contacts^4,5^ and quantum dot devices that hold charge or spin qubits^6–12^. By coupling the quantum devices to lumped-element electrical resonators or on-chip stub tuners^13^, it is possible, in principle, to perfectly match the impedance of the devices to the RF feedlines connected to them^14^. This ensures an optimum power transfer to the devices, and the best charge and capacitance sensitivities are expected in this read-out regime^15^. However, in practice there are often large and unpredictable variations between device impedances. It is therefore necessary to have in situ tunability of the resonator circuits to ensure perfect matching. In addition, tunability of the resonant frequencies of the circuits is required to achieve optimal performance when using RF components that have a narrow operation bandwidth (such as many low-noise amplifiers)^16^ or for multiplexing signals of several read-out channels^17^.

In situ tunability for impedance matching and frequency tuning can be achieved using voltage-tunable capacitors, which are also known as varactors. However, commercially available varactors are not designed for low-temperature operation. Semiconductor-based varactors have been shown to work down to temperatures of around 1 K (refs. ^18–22^), but their performance degrades substantially in the millikelvin temperature range, at which most solid-state quantum devices are operated due to the freezing out of free charge carriers.

Another type of varactor is based on ferroelectric materials, which have a high dielectric permittivity that can be tuned by an electric field. Ferroelectrics in the lead titanate and barium strontium titanate (BST) families are often used. BST (Ba_1−*x*_Sr_*x*_TiO_3_) is a particularly well-studied material with good tunabilty at room temperature and relatively low loss tangent. These materials are, however, typically operated in their paraelectric state—that is, well above their Curie temperature—to minimize losses. At low temperatures, materials such as BST become ferroelectric and lose their tunability, and dissipative losses increase. Some materials—including strontium titanate (SrTiO_3_), potassium tantalate (KTaO_3_) and calcium titanate (CaTiO_3_)—can, though, exhibit quantum paraelectricity, a phenomenon where ferroelectric order is suppressed by quantum fluctuations down to zero kelvin.

In this Article, we show that quantum paraelectric materials can be used to make varactors that operate at millikelvin temperatures. We focus on SrTiO_3_ varactors because the material has a very high relative permittivity (on the order of 10,000) at temperatures below ∼4 K, which can be tuned by over an order of magnitude using an electric field in this temperature range^23–27^. We also show that KTaO_3_ can be used to make millikelvin varactors, though the electric field tunability is lower than for SrTiO_3_. We show that SrTiO_3_ varactors can be easily fabricated and allow frequency tuning and perfect impedance matching down to 6 mK when integrated into an RF measurement circuit. The performance of the varactors at 6 mK (the lowest achievable temperature in our measurement system) is characterized in terms of their capacitance tunability, dissipative losses and magnetic field dependence. To illustrate their use in RF read-out, we measure single quantum dot (SQD) and double quantum dot (DQD) devices and show that the SrTiO_3_ varactors allow optimization of the signal-to-noise ratios (SNRs) to achieve charge and capacitance sensitivities of 4.8 μ*e* Hz^−1/2^ and 0.04 aF Hz^−1/2^, respectively.

## Matching network and device

**Fig. 1:**
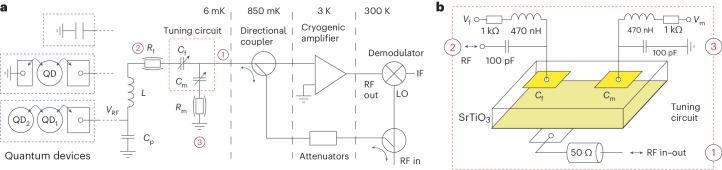
Schematic of the RF detection circuit. **a**, In different experiments, a fixed capacitance, an SQD and a DQD device are embedded in an *LC* resonant circuit for high sensitivity and high bandwidth measurements. Two SrTiO_3_ varactors are incorporated in the circuit for frequency tuning and impedance matching. The RF signals are amplified using a low-noise cryogenic amplifier operating at 3 K. Room temperature demodulation provides a measurement of both RF quadratures. **b**, The two varactors are fabricated on a single SrTiO_3_ crystal and have a parallel plate geometry. The varactors are voltage biased using bias tees and characterized in the schematic in **a** by a variable capacitance and effective series resistance to incorporate losses. IF, intermediate frequency; LO, local oscillator.

The RF detection circuit we consider is shown schematically in Fig. 1a. It consists of a quantum device coupled to a resonant circuit formed by an inductor *L* and parasitic capacitance *C*_p_. Two SrTiO_3_ varactors are included for frequency tuning and impedance matching, as illustrated in Fig. 1b. The varactors consist of parallel metallic pads (5/60 nm Ti/Au) on either side of a 0.5 mm TiO_2_-terminated single-crystal SrTiO_3_ (001) substrate. Top pads of varying dimensions were fabricated using optical lithography—the smallest of circular shape and 50 μm diameter—that are voltage biased against the fully metallized bottom of the substrate. The latter is directly connected to the RF measurement line. In our experiments, the two varactors are either fabricated on separate SrTiO_3_ substrates or, as in Fig. 1b, on the same 3 × 3 mm^2^ substrate and separated by about 2 mm. In this work, we first characterize the varactors by coupling the circuit to a known fixed capacitor and then use the detection circuit for the read-out of SQD and DQD devices relevant for quantum information processing.

The circuits are measured in a dilution refrigerator with a base (lattice) temperature of 6 mK. All d.c. lines are filtered at various stages with a filter bandwidth of ∼3 kHz. We use two separate d.c. control voltages, *V*_f_ and *V*_m_, applied via bias tees, to tune the two varactors as indicated in Fig. 1b. The RF signal is attenuated at various stages along the dilution refrigerator and applied to the device and matching circuit using a directional coupler. The reflected signal is amplified using a low-noise amplifier with noise temperature *T*_N_ ≈ 5 K mounted at the 3 K stage and demodulated at room temperature. In measurements on DQDs (discussed below), we have additionally incorporated a Josephson parametric amplifier (JPA) mounted at the mixing chamber stage of the dilution refrigerator.

## Impedance matching and frequency tuning

**Fig. 2:**
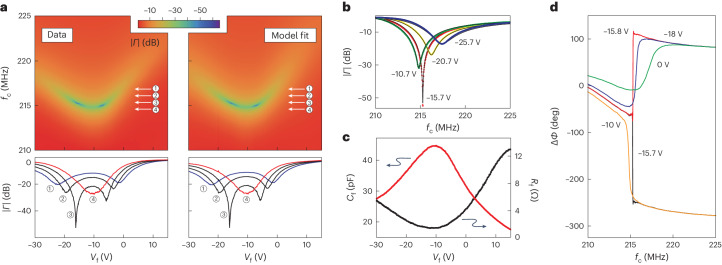
SrTiO_3_ varactor characterization. **a**, Colourscale plot of the measured reflection coefficient magnitude ∣*Γ*∣ (left) and model fits (right) as a function of RF frequency and varactor voltage *V*_f_. For these measurements *V*_m_ is set to zero. The data shows shifts of the resonance frequency, reaching a minimum around *V*_f_ ≈ −10 V. The simultaneous change in matching conditions results in a strong variation of the measured magnitude. Perfect matching is observed around *V*_f_ = −15.7 V and −7.0 V. The linegraphs show the reflection coefficient magnitude ∣*Γ*∣ as a function of varactor voltage along the directions indicated by the arrows. **b**, Reflection coefficient magnitude ∣*Γ*∣ data and model calculations (solid lines) as a function of frequency for several different varactor voltages as indicated. **c**, Effective capacitance and resistance values for the varactors obtained from the data fits in **a**, showing a maximum *C*_f_ of 45 pF. **d**, Measured RF phase response as a function of frequency for several different varactor voltages as indicated. The data show clear transitions from under- to over-coupling, with perfect matching observed here around *V*_f_ = −15.7 V.

We first characterize the SrTiO_3_ varactors by measuring the response when the detection circuit is coupled to a fixed capacitance of ∼2.8 pF as in the top left inset in Fig. 1a. Figure 2a shows the resulting RF amplitude response as a function of frequency and control voltage *V*_f_. Here we set *V*_m_ = 0 V and vary *V*_f_ between −30 V and 15 V. This results in a pronounced shift of the resonance frequency, reaching a minimum of ∼215 MHz at *V*_f_ = −10 V and a maximum of ∼223 MHz at *V*_f_ = 15 V. At the same time, the strength of the amplitude response changes, as also seen in the line scans of Fig. 2a, demonstrated a simultaneous change in the matching conditions. Matching is observed around *V*_f_ = −15.7 V and −7.0 V with the device being over-coupled to the RF feedline within this range and under-coupled elsewhere.

To quantitatively understand the response of the SrTiO_3_ varactors, we parameterize the varactors by their voltage-dependent capacitance and effective series resistance, which takes into account dissipative losses. Using these two variables as fit parameters, we are able to obtain excellent agreement with the observed data (see the right column of Fig. 2a and Supplementary Information Section I for further details on the fit procedure). The results are summarized in Fig. 2c, which shows the values obtained for the frequency tuning varactor capacitance *C*_f_ and effective series resistance *R*_f_ as a function of the control voltage *V*_f_. Over the voltage range measured, the capacitance can be tuned between 18 pF and 45 pF, with the maximum value observed at *V*_f_ = −10 V. The effective series resistance has a minimum of about 1.5 Ω when the capacitance is at a maximum. The resistance values include contributions from the chip inductor and circuit board and so should be considered an upper bound for the losses in the varactor. Similar tunability is observed for the second varactor in the circuit, controlled by *V*_m_. Operating the two varactors together allows independent tuning of the resonance frequency and matching conditions.

These measurements are consistent with the behaviour of SrTiO_3_ at higher temperatures and show that the material retains its characteristic tunability down to millikelvin temperatures. Using finite-element simulations of our device geometry we obtain a relative permittivity of approximately 20,000 for the maximum capacitance of 45 pF observed here, in good agreement with expectations for SrTiO_3_ (ref. ^28^). The extracted values for the capacitance and effective series resistance also allow us to estimate the dielectric loss tangent of the varactor defined as tan$(\delta )={\epsilon }^{{\prime\prime} }/{\epsilon }^{{\prime} }$, where ${\epsilon }^{{\prime\prime} }$ and ${\epsilon }^{{\prime} }$ are the imaginary and real part of the relative permittivity^14^. The loss tangent can be related to the effective series resistance tan(*δ*) = *R*_f_*C*_f_ *ω*, where *ω* = 2π*f* is the angular frequency of the measurement. Because in our experiments we cannot distinguish between loss contributions from the SrTiO_3_ varactors and the chip inductor and circuit board, it is difficult to provide an exact value for the varactor loss tangent. Nevertheless, from the values for *R*_f_ and *C*_f_ extracted in Fig. 2c, we can estimate tan(*δ*) to be in the 10^−1^–10^−2^ range. Dissipation in SrTiO_3_ has been attributed to the interaction between the oscillating (RF) electric field and acoustic phonons, where the presence of a large d.c. electric field breaks the crystal lattice symmetry and introduces a field-dependent dipole moment in the unit cell, making the system more susceptible to RF losses^29^. In single crystals, this results in an increase of the observed loss tangent for larger d.c. electric fields, which is consistent with our data.

The shift of the maximum capacitance to negative voltages, as seen in Fig. 2c, has previously been reported (for example, in ref. ^30^) and has been attributed to trapped charges, such as oxygen vacancies, in the crystal. This also creates hysteretic behaviour and a dependence of the capacitance on the voltage sweep history. We verified that this does not affect device stability by measuring the phase and magnitude response at the RF resonance frequency over time. More details on the observed hysteresis and varactor stability at millikelvin temperatures are provided in the Supplementary Information Section IV.

## Optimizing signal-to-noise ratio

The ability to tune the impedance of the RF reflectometry read-out set-up with the varactor tuning circuit allows optimization of the measured SNRs and therefore of the achievable charge or capacitance sensitivity. This can be understood by considering the expected SNR:

1 $${{{\rm{SNR}}}}={\left\vert \frac{\partial \varGamma }{\partial X}\Delta X\right\vert }^{2}\frac{{P}_{\rm{C}}}{{P}_{\rm{N}}}$$

where Δ*X* can be either Δ*R*, a change in the effective device resistance, or Δ*C*, a change in device capacitance (or potentially both) and *P*_C_ and *P*_N_ are the applied RF carrier power at the input of the tuning circuit and the noise power added to the signal, respectively^15^. In our measurement set-up, the noise is dominated by the low-temperature amplifiers in the amplification chain and can be expressed as *P*_N_ = *k*_B_*T*_N_Δ*f*, where *k*_B_ is the Boltzmann constant, *T*_N_ is the noise temperature of the amplifier and Δ*f* is the equivalent noise-power measurement bandwidth.

**Fig. 3:**
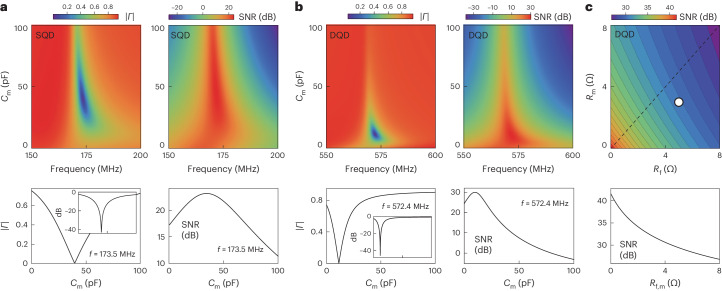
Modelled RF measurement response as a function of varactor capacitance. **a**, Left, calculated reflectance magnitude ∣*Γ*∣ as a function of frequency and *C*_m_ for the SQD device using the circuit described in the text. The line graph shows the corresponding trace at a frequency *f* = 173.5 MHz (inset, same graph on decibel scale). Right, calculated SNR as a function of frequency and matching capacitance *C*_m_ for the SQD device using the experimental parameters and *P*_C_ = −110 dBm. **b**, Left, calculated reflectance magnitude ∣*Γ*∣ as a function of frequency and matching capacitance *C*_m_ for the DQD device using the circuit described in the text. The line graph shows the corresponding trace at a frequency *f* = 572.4 MHz (inset, same graph on decibel scale). Right, calculated SNR as a function of frequency and matching varactor capacitance *C*_m_ for the DQD device using the experimental parameters and *P*_C_ = −105 dBm. **c**, Contour plot showing the maximum SNR observed in the DQD SNR plots calculated for a range of the effective series resistances *R*_m_ and *R*_f_ between 0 and 8 Ω. The line scan shows the SNR along the dashed black line in the contour plot. The white dot indicates the varactor resistance values used in the calculations.

The expected response for the reflection coefficient magnitude ∣*Γ*∣ and the SNR as a function of frequency and matching varactor capacitance is shown in Fig. 3a for the experimental parameters and frequency range relevant for the SQD device and read-out circuit (and similarly in Fig. 3b for the DQD device). Here we kept the effective varactor resistance values fixed but show in Fig. 3c how the SNR changes as these values vary. Two important qualitative features are apparent in these model calculations that show how device read-out depends on the matching varactor capacitance *C*_m_. First, the reflection coefficient magnitude ∣*Γ*∣ is sensitively dependent on *C*_m_ and impedance matching is only achieved around a narrow range of the varactor capacitance. As values of stray capacitances are hard to predict a priori, the ability to tune the varactor capacitance *C*_m_ in situ is key for obtaining perfect impedance matching or to tune the device into a desired over- or under-coupled regime. Second, the SNR can be optimized by tuning the device towards impedance matching to maximize ∂*Γ*/∂*X* in equation (1), that is, the tuning optimizes the sensitivity of the reflection coefficient with respect to small changes in the device parameters Δ*R* or Δ*C* (ref. ^31^). For the circuit parameters used here, which aim to model the circuit and quantum dot devices described below, the improvement of the SNR when *C*_m_ is tuned to its optimum value is about 4–6 dB when compared to having no matching varactor in the read-out circuit (the SNR value for *C*_m_ = 0). This is seen in the rightmost plots of Fig. 3a,b. The improvement in the SNR can be increased further by reducing the varactor losses, as shown in Fig. 3c. Efforts to this effect are described in Conclusions.

We further note that minimizing the background reflectance as the device is tuned towards impedance matching has the additional important benefit that it enables us to optimize the use of ultra-low-noise amplifiers, such as JPAs, in the amplification chain. This type of amplifier is easily saturated (typically around input powers of −120 dBm for a gain of 20 dB). Minimizing ∣*Γ*∣ using the varactors allows us to increase the threshold carrier power that can be applied before the JPA saturates, therefore maximizing *P*_C_/*P*_N_ in equation (1) and thus further improving the SNR.

## SQD charge sensitivity

**Fig. 4:**
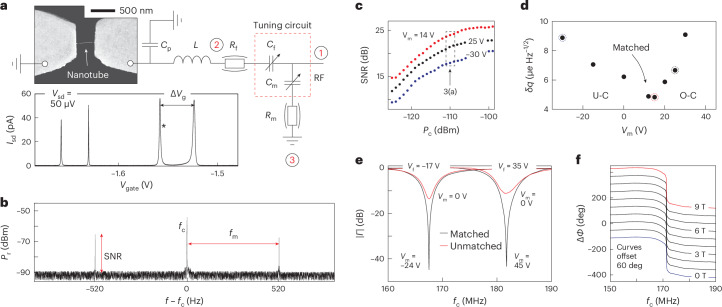
SQD RF measurements. **a**, Simplified schematic of the RF detection circuit. The scanning electron micrograph shows the carbon nanotube quantum dot device embedded in the circuit. The inset shows the measured d.c. current *I*_sd_ between the source and drain electrode as a function of back-gate voltage *V*_gate_ for a bias voltage *V*_sd_ of 50 μV applied using a bias tee not shown in the schematic. **b**, Reflected power (*P*_r_) spectrum close to matching showing the RF carrier signal at *f*_c_ and sidebands resulting from a back-gate voltage modulation with frequency *f*_m_ = 520 Hz. The SNR is obtained from the height of the sidebands with respect to the noise background as indicated. **c**, SNR as a function of carrier power for different settings of the varactor *V*_m_. **d**, Charge sensitivities as a function of *V*_m_. The three highlighted points correspond to the SNR curves of **c** measured at −99 dBm. The read-out is tuned from the under-coupled (U-C) to the over-coupled (O-C) regime through perfect impedance matching. The best sensitivities are observed here for *V*_m_ ≈ 14 V when the device is impedance matched. **e**, The resonance frequency and impedance matching can be independently tuned using appropriate combinations of *V*_f_ and *V*_m_ as indicated. **f**, Phase response as a function of magnetic field with the varactors tuned to impedance matching. No change is observed in the resonance frequency or matching over a range of 9 T.

To demonstrate the relevance of impedance matching using SrTiO_3_ varactors for improving RF read-out, we measure the charge sensitivity of a carbon nanotube quantum dot at *T* = 6 mK, as shown in Fig. 4a. For these measurements we set the back-gate voltage to the steepest slope of a conductance peak (indicated by the asterisk in the inset of Fig. 4a) and modulate the back-gate voltage with a small sinusoidal voltage *V*_rms_ = 10 μV and modulation frequency *f*_m_ = 520 Hz. This gate modulation and corresponding changes in the nanotube resistance Δ*R* cause an amplitude modulation of the carrier signal. In the power spectrum of the reflected signal shown in Fig. 4b, this results in sidebands at carrier frequency *f*_c_ ± *f*_m_. The SNR is then determined from the height of the sidebands with respect to the noise floor and measured for a range of varactor voltages and RF carrier powers.

The results of these measurements are illustrated in Fig. 4c, which shows the SNR as a function of applied carrier power for three different settings of the matching varactor with *V*_m_ ≈ 14 V, the voltage corresponding to perfect impedance matching for this measurement. For all *V*_m_ curves, the SNR first increases with increasing carrier power, consistent with equation (1). For large applied powers, the SNR decreases again due to the onset of nonlinearity of the device resistance at a conductance peak and the resulting power broadening by the RF carrier signal^12^. The results are consistent with the expected effect of the varactor to change the ∂*Γ*/∂*R* term in equation (1), which, on a logarithmic decibel scale, appear as fixed offsets for different *V*_m_ independent of carrier power, as seen in Fig. 4c.

The SNR results in the linear-response regime can also be compared to the simulations for this device shown in Fig. 3a, which model the SNR response for an RF carrier power *P*_c_ = −110 dBm and resistance modulation Δ*R* = 500 kΩ at the Coulomb resonance. Although a precise quantitative comparison is complicated by the simultaneous change of the varactor capacitance and effective resistance with *V*_m_, both the absolute values of the SNR observed at *P*_c_ = −110 dBm (indicated by the arrow in Fig. 4c) and the SNR change with varactor voltage are in good agreement with the model calculations. More specifically, the experimental results show that the SNR improves by about 4–6 dB by tuning the device towards impedance matching using the varactors.

The importance of being able to tune the resonator matching conditions is also apparent in Fig. 4d where we plot the corresponding results for the charge sensitivity *δq*, which is related to the SNR using the following relation^3^:

2 $$\delta q=\frac{\Delta {q}_{\rm{rms}}}{1{0}^{{{{\rm{SNR}}}}/20}\sqrt{2\Delta f}}$$

where Δ*f* = 3.4 Hz is the resolution bandwidth used here and Δ*q*_rms_ is the gate charge induced by the oscillating voltage on the back gate. The latter is obtained from *V*_rms_ and the known gate capacitance *C*_g_ = *e*/Δ*V*_g_, where *e* is the electron charge and Δ*V*_g_ is the gate voltage difference between two Coulomb blockade conductance peaks as shown in the graph of Fig. 4a. For our carbon nanotube quantum dot, this yields Δ*q*_rms_ = 2.4 × 10^−4^*e*. Each data point in Fig. 4d corresponds to the charge sensitivity obtained for a different varactor voltage *V*_m_ and a carrier power *P*_c_ = −99 dBm, which is the approximate power at which the SNR peak occurs (Fig. 4c). We observe an excellent minimum charge sensitivity *δq* ≈ 4.8 μ*e* Hz^−1/2^ when the device is tuned towards impedance matching at *V*_m_ ≈ 14 V.

## Frequency tuning and magnetic field insensitivity

For operation in a reflectometry set-up, it is important to be able to tune both the resonant frequency and impedance matching independently. This ability is demonstrated in Fig. 4e where we operate both of the SrTiO_3_ varactors. We first use *V*_f_ to set the resonant frequency, followed by applying a suitable voltage on *V*_m_ to obtain perfect matching. Using the two varactors, we are able to set both the resonant frequencies and obtain impedance matching in a frequency window between 167 MHz and 182 MHz for control voltages *V*_f_ and V_m_ < 50 V. The obtained frequency shift is larger than the bandwidth of the resonances, which is relevant in applications for which multiplexing is important. Additionally, we tested the magnetic field dependence of the SrTiO_3_ varactors as shown in Fig. 4f for the phase response. No change is observed over the measurement range up to 9 T for both the resonance frequency and matching. This is important for applications in which a magnetic field is used to tune device characteristics, such as spin-based quantum information processing. Using SrTiO_3_ varactors, any variation in the measured amplitude or phase can then be confidently attributed to the tested devices rather than changes in the matching or detection circuit.

## DQD capacitance sensitivity

**Fig. 5:**
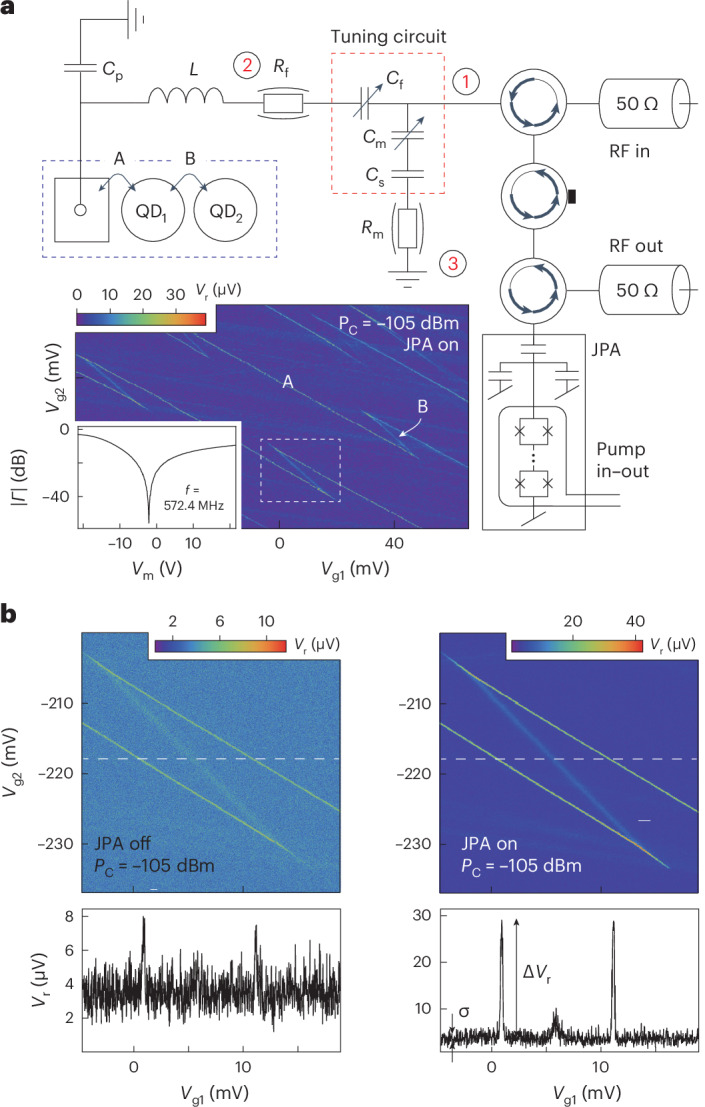
DQD RF measurements. **a**, Simplified schematic of the RF detection circuit of the DQD device, which includes a JPA as the first-stage amplifier. The inset shows the measured DQD charge stability diagram as a function of plunger gate voltages *V*_g1_ and *V*_g2_. The line trace shows the measured reflectance magnitude ∣*Γ*∣ as a function of matching varactor voltage *V*_m_ for a frequency *f*_c_ = 572.4 MHz measured in a regime where transport is Coulomb blockaded. **b**, Part of the DQD stability diagram as indicated by the white dashed lines in **a**. The left plot shows the measured signal amplitude *V*_r_ for *P*_C_ = −105 dBm with the JPA off (not pumped), and the right plot shows the same measurement with the JPA on (pumped), which greatly increases the SNR. The line traces show *V*_r_ as measured along the white dashed lines in the figures.

The varactors also allow optimization of RF reflectometry read-out of DQD devices, which are of interest as charge or singlet–triplet spin qubits. To demonstrate this, we measured a carbon nanotube DQD coupled to a single source electrode and controlled by two separate gate electrodes as illustrated in Fig. 5. For a DQD geometry, charge transitions—for example, the ability of an electron to move between the source and left quantum dot, or between quantum dots, in response to an applied voltage—can be understood, or modelled, as an effective (quantum) capacitance Δ*C* rather than resistance, assuming the tunnel rate is larger than the RF drive frequency^7^. In Fig. 5a charge transitions between the source electrode and left quantum dot are labelled ‘A’ and those between the two quantum dots are labelled ‘B’.

For the RF reflectometry measurements on the DQD device, we used the same varactors as for the SQD device. However, as the DQD device has been fabricated on an undoped Si/SiO_2_ substrate, its parasitic capacitance *C*_p_ = 0.27 pF is substantially lower as compared to that of the SQD device where it is dominated by the degenerately doped substrate. We furthermore used a different chip inductor with inductance *L* = 220 nH, yielding a resonance frequency in the 570–580 MHz range. These values were chosen such that the resonator frequency is compatible with the use of a JPA additionally incorporated in the set-up, which operates in this frequency range^16^. As is evident from the simulations in Fig. 3b, to tune the device through matching now requires a matching capacitance in the 5–15 pF range. To achieve this with the same varactors as used for the measurements on the SQD device, we additionally placed a fixed *C*_s_ = 20 pF capacitor in series with the matching varactor such that the effective *C*_m_ is reduced accordingly. This allows impedance matching (inset of Fig. 5a) in which the resonator response was measured at a frequency of 572.4 MHz and tuned using *V*_m_. We note that in these experiments the importance of the varactors is two-fold: to optimize ∂*Γ*/∂*C* and to minimize the background reflectance, allowing us to maximize the *P*_C_/*P*_N_ ratio when incorporating ultra-low-noise JPAs in the amplification chain.

The optimization of our read-out using the varactors allows fast read-out and state-of-the-art capacitance sensitivities, which is required for quantum information processing applications. This is demonstrated in Fig. 5 by using a filter time constant on the demodulation circuit of 1 μs such that stability diagrams are obtained on millisecond timescales. For these ‘video mode’ measurements^32^, rather than using sidebands in the power spectrum (as in Fig. 4), we instead extract the SNRs from the measurement of ${(\Delta {V}_{\rm{r}}/\sigma )}^{2}$ where Δ*V*_r_ is the measured voltage at the charge transition with respect to the background signal and *σ* the standard deviation of the background when the device is Coulomb blockaded (see line scans in Fig. 5b). We obtain an SNR of 14 dB for transition A without the JPA (left plot) and an SNR of 29 dB with the JPA (right plot). The SNR improvement of 15 dB with the JPA is as expected, given the difference in noise temperature: *T*_N_ = 5 K without the JPA and *T*_N_ = 0.15 K with the JPA (Supplementary Information Section I).

The obtained 29 dB SNR at matching is also consistent with model calculations of the device in Fig. 3b. Here we modelled transition A using a capacitance Δ*C* = 0.4 fF. This value follows from the relation Δ*C* = (*e*^2^*α*^2^)/(4*k*_B_*T*_e_), where *α* = 0.1 ± 0.02 is a lever arm obtained from the stability diagram that relates voltages on the electrodes to changes in electrochemical potential of the quantum dot and *T*_e_ = 12 mK is the electron temperature of the device, which is obtained from using the quantum dots as a primary thermometer. From the measured SNR, we can calculate the obtained capacitance sensitivity:

3 $$\delta C=\frac{\Delta C}{1{0}^{{{{\rm{SNR}}}}/20}\sqrt{2\Delta f}}$$

where we use the quantum capacitance Δ*C* = 0.4 fF, as described above, and include Δ*f* = 78 kHz, the equivalent noise-power bandwidth for our read-out (see Supplementary Information Section I for details). For the measured SNR of 29 dB this yields a state-of-the-art *δC* = 0.04 aF Hz^−1/2^. Here, the RF carrier power *P*_C_ = −105 dBm was chosen to optimize the SNR without resulting in any major power broadening, yielding an estimated RF voltage across the transition of ∼4–6 μV. To put the capacitance sensitivity into context, the states of a singlet–triplet qubit can be distinguished by their quantum capacitance, which is typically zero for the triplet state and finite for the singlet state. If we assume a conservative Δ*C* = 0.1 fF for the singlet, the capacitance sensitivity achieved here implies single-shot (SNR = 1) spin state read-out on a timescale of about 225 ns, which is many orders of magnitude shorter than typical spin coherence or relaxation times.

## Conclusions

We have reported that SrTiO_3_ varactors can work effectively at the millikelvin temperature range and can be used in RF reflectometry of solid-state quantum devices. The ability of varactors to tune resonator frequencies in situ allows for scalable multiplexed quantum architectures, reducing the number of connections and wiring needed. At the same time, varactor impedance matching greatly improves read-out sensitivity, as we demonstrated for the read-out of SQD and DQD devices. While using the varactors resulted in an improvement in the measured SNR by about 4–6 dB, the model calculation shown in Fig. 3c indicates there is the potential for even better performance if varactor losses in the detection circuit can be reduced.

One option is to replace SrTiO_3_ with potassium tantalate (KTaO_3_)^33,34^. The quantum paraelectric KTaO_3_ has a loss tangent predicted to be two orders of magnitude smaller than that of SrTiO_3_ at temperatures below 1 K, at the expense of a somewhat lower electric-field tunability^35^. Supplementary Information Section III shows our millikelvin measurements on KTaO_3_ varactors. It shows KTaO_3_ does have a lower electric-field tunability compared to SrTiO_3_ but also has similar losses, which is not currently fully understood. One possibility is that edge effects or surface defects at the electrode interface might be more important for the varactors used in our work when compared to much larger KTaO_3_ specimens studied previously, which are typically of millimetre size. Further study of KTaO_3_ varactors at millikelvin temperatures of different sizes, geometries and crystal orientations would be of value.

Finally, we note that our work is readily extendable to other capacitance or frequency ranges. For example, the parallel plate varactor geometry used here shows capacitance tunability between about 45 pF and 15 pF, but the design could be customized for different capacitance values using different area sizes or interdigitated electrodes. We expect the varactors could be made to operate in the gigahertz frequency range, ultimately being limited by the soft optical mode phonon frequency^36^.

## Methods

### SrTiO_3_ and KTaO_3_ varactor fabrication

The back sides of 3 × 3 mm^2^ single-crystal (001) TiO_2_-terminated strontium titanate or single-crystal (100) potassium tantalate substrates (all 0.5 mm thick, single-side polished, purchased from SurfaceNet) are metallized with 5/60 nm of Ti/Au. The top (polished) sides of the substrates are coated with a double layer of photoresist: LOR10B (∼1 μm thick, baked at 190 °C for 10 min) and S1805 (∼0.5 μm thick, baked at 115 °C for 1 min). We have used both quartz-chrome photomasks and direct write photolithography systems to expose pads of various sizes on the photoresist, varying from 50 to 150 μm, typical dimensions. For the quantum dot measurements in the main text, we used square pads of 120 × 120 μm^2^. The substrates are developed in MF-26A for 45 s and the pads metallized with 5/60 nm of Ti/Au. The devices are then sonicated in Microposit 1165 remover in a heat bath at 80 °C for lift off.

Both the frequency-tuning and impedance-matching varactors used in the quantum dot measurements are fabricated on the same substrate and are separated by about 2 mm. The back side of the varactors substrates are directly connected to a printed-circuit board (PCB) using silver paste and annealed for 5 min at 120 °C to ensure good conductance. The varactor pads on the top side are bonded to the PCB using Au wires of 25 μm diameter. The PCB is enclosed in a brass sample holder connected to a cold finger at the mixing chamber of a dilution refrigerator. Subminiature push-on connectors on the PCB are used to connect to the coaxial measurement lines.

### Carbon nanotube device fabrication

Single-wall carbon nanotubes were grown by chemical vapour deposition on degenerately doped SiO_2_ substrates terminated with a 280 nm dry thermal oxide. The room temperature resistivity of the Si wafers for the SQD and DQD devices is *ρ* < 1.5 mΩ cm and *ρ* > 4,000 Ω cm, respectively. The carbon nanotubes were distributed at a concentration of approximately one nanotube per 20 μm^2^ on the substrate. Device fabrication consisted of two electron-beam lithography and metal evaporation stages using 5% (by weight) of 495K polymethyl methacrylate dissolved in anisole as a resist. During the first stage, alignment marks and bond pads were fabricated on the substrates using 5/60 nm of Ti/Au. The carbon nanotubes were subsequently located with respect to the alignment marks using atomic force microscopy. During the second lithography stage, the source and drain electrodes were defined using 5/60 nm of Ti/Au. A wedge bonder is used to connect the various bond pads of the device to the sample holder using Au wires of 25 μm in diameter.

### Experimental methods

Experiments were carried out in a dry dilution refrigerator with a base temperature of 6 mK. The measurement circuit included several d.c. lines which were thermally anchored and extensively filtered at various temperature stages with a d.c. filter time constant of ∼3 kHz, yielding a carbon nanotube electron temperature of ∼12 mK. The RF detection circuit was connected to the device, as shown in the schematics in the main text. Bias tees allowed for both RF and d.c. signals to be applied to the device source electrode and varactors (see Supplementary Information Section II for a photograph of the assembled circuit). The reflected RF signals were measured with a vector network analyser and a high-frequency lock-in amplifier (Zurich Instruments UHFLI). Further details are provided in Supplementary Information Section I.

## Supplementary information

Supplementary Information Supplementary Sections I–V and Figs. 1–5.

## Data Availability

The data that support the findings of this study are available via Zenodo at 10.5281/zenodo.12511105 (ref. ^37^).
