# Supplementary material for: Quantum paraelectric varactors for radiofrequency measurements at millikelvin temperatures
Source: Nat Electron. 2024 Aug 5;7(9):760–7. doi: 10.1038/s41928-024-01214-z (PMC11422161; doi:10.1038/s41928-024-01214-z)
Supplement: Supplementary file 1 — Supplementary Sections I–V and Figs. 1–5. [file 41928_2024_1214_MOESM1_ESM.pdf]

nature electronics

Supplementary information https://doi.org/10.1038/s41928-024-01214-z

Quantum paraelectric varactors for radiofrequency measurements at millikelvin temperatures

In the format provided by the authors and unedited

I MATCHING NETWORK SIMULATION

The $SrTiO_3$ parallel plate varactors measured in Fig. 2 in the main text were fabricated on two separate 3x3 mm single-crystal (001) TiO_2 substrates with pads of 100 μm diameter each. The detailed circuit model used to simulate the $SrTiO_3$ varactors response are shown in Supplementary Figure 1. The two $SrTiO_3$ varactors are modelled as lumped elements with capacitance values C_m and C_f which are tuned by the control voltages V_m and V_f, respectively. Dissipative losses are modelled by the resistors R_m and R_f. The control voltages are applied via bias tees that consist of a 10 kΩ resistor, a 220 pF coupling capacitor and 470 nH inductor. A further bias tee is included that allows the application of a source-drain bias voltage V_{sd} over the device. For the $SrTiO_3$ varactors characterisation measurements, a fixed 2.8 pF capacitor is included in the circuit (rather than a quantum dot device).

To take into account the self capacitance of the inductors, these were modelled to be parallel with a capacitance and resistor as indicated by the rightmost panel in Supplementary Figure 1. The values for C_L and R_C were taken from the inductor datasheets (model: Coilcraft 0805CS-221): L=220 nH, R_c=26 Ω and C_L=0.086 pF and (model: Coilcraft 0805CS-471): L=470 nH, R_c=66 Ω and C_L=0.132 pF. The datasheets also include values for frequency-dependent series resistors that model wire losses (of order 6-8 Ω at the frequencies of interest). We were unable to fit the measured data with these values and suggest that the datasheet - obtained at room temperature - overestimates the values of the series resistors when measured at 6 mK. Instead we modelled the dissipative losses of the 220 nH inductor by incorporating this in the fit parameter R_f. For the 470 nH bias tee inductors these values are not significant for our model as the inductors are in series with 10 kΩ resistors.

Using the model we calculate the reflection coefficient Γ given by [1]:

$$\Gamma(f) = \frac{Z(f) - Z_0}{Z(f) + Z_0} \tag{1}$$

where $Z(f)$ is the total impedance of the network in Supplementary Figure 1 for a given frequency f and Z_0=50 Ω is the line impedance to which it is connected. The reflection coefficient is measured using a vector network analyzer where we take into account a constant insertion loss resulting from attenuation in the measurement lines and amplifier gains. In our measurement set-up, the total attenuation in the measurement lines amounted to 85 $\pm$ 4 dB: 40 dB from fixed attenuators at room temperature and 45 dB in our dilution refrigerator which includes fixed attenuators at various temperature stages, a directional coupler and losses in the coaxial lines. Amplification in the return line is provided by a cryogenic amplifier operated at 3 K, providing 45 dB gain, and a further amplification stage at room temperature providing 60 dB gain. For a perfectly matched network the reflection coefficient is zero. In practice the minimum detectable signal amplitude is limited by the noise floor of the network analyzer.

The data fits in Fig. 2a in the main text were obtained from the expression for Γ above using C_f and R_f as fit parameters for all frequency sweeps, as V_f was varied. Other parameters, including an overall offset to account for the

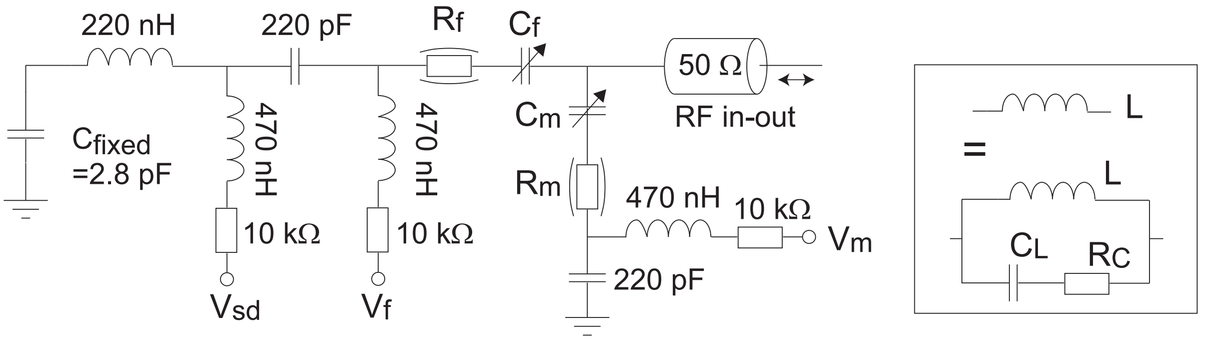

Supplementary Figure 1. Circuit model used to simulate the $SrTiO_3$ varactors response. The varactors are taken as lumped elements with voltage tunable capacitances C_f, C_m and effective series resistances R_f, R_m, respectively. To take into account the self capacitance of the inductors, these were modelled to be parallel with a capacitance and resistor as indicated by the rightmost panel.

insertion loss, were kept fixed. We used Mathematica's FindFit routine (least-squares fit) for each line fit. Relevant fitting constraints were introduced to ensure the fitting parameters made physical sense. In particular we ensured that the fit parameters were in the correct under or overcoupled regime as obtained from the phase response. That is, close to matching, fits to the amplitude generally provided good results for both slightly undercoupled and overcoupled impedances and comparison of the phase response of the simulations and the data allowed us to differentiate between the two regimes.

The circuit models we considered for the single (SQD) and double quantum dot (DQD) simulations (Fig. 3 in the main text) are shown in Supplementary Figure 2. The main difference between the circuits for the two experiments are the values of the parasitic capacitance and inductor used as indicated in the figure. For the SQD measurements the parasitic capacitance is dominated by the bond pads on the degenerately doped Si/SiO_2 substrate and is therefore relatively large. For the SQD measurement we used (model: Coilcraft 0805CS-331): L=320 nH, R_c=31 Ω and C_L=0.096 pF. The (model: Coilcraft 0805CS-221): L=220 nH, R_c=26 Ω for the DQD measurement was chosen such that the resonator frequency was with the approximately 550-600 MHz operational range of our JPA and cryogenic circulators and isolators.

As shown in the simulations of Fig. 3 in the main text, the optimum matching varactor capacitance to tune the device towards impedance matching is in the 30-50 pF range for the SQD measurements and in the 5-15 pF range for the DQD measurements. We used the same varactors in both experiments and were able to tune the device to impedance matching in both cases, but to achieve this for the DQD measurements required us to include a fixed 20 pF capacitor in series with the matching varactor as indicated in Supplementary Figure 2. Including a fixed capacitor has the advantage of reducing the overall capacitance to a desirable setpoint while maintaining tuneability, albeit over a smaller capacitance range. Also note that for the simulations we kept R_m fixed as C_m is varied, while in practice both variables will change in a measurement as seen in Fig. 2c in the main text. While this does not qualitatively affect the simulated varactor response, the resistance values do have an effect on the SNR that is achieved - and correspondingly some uncertainty in the modelled SNR values. The dependence of the SNR on both R_f and R_m are shown in Fig. 3c in the main text.

For the SNR simulations we used a noise power $P_N = k_B T_N \Delta f$. In our set-up, the noise for the SQD measurements (Fig. 4 in the main text) is dominated by the low-temperature amplifier (CITLF1) mounted at the 3K stage of the dilution refrigerator which has a noise temperature $T_N = 5$ K. For the DQD measurements an additional JPA mounted at the dilution refrigerator mixing chamber plate was used [2]. For those measurements (Fig. 5 in the main text) the SNR improvement of $\sim$ 15 dB is as expected given the difference in noise temperature: $T_N = 5$ K without the JPA and $T_N = 0.15$ K with the JPA. The latter consists of the noise temperature of 0.1 K of the JPA itself and the residual contribution of the (now) second-stage cryogenic amplifier with noise temperature 5 K divided by the 20 dB gain of the JPA. For the DQD readout we used a Zurich Instruments UHFLI with time constant $\tau = 1$ μs and filter order four (roll-off 24 dB/oct) yielding a noise-power equivalent bandwidth $0.0781/\tau = 78.1$ kHz [3].

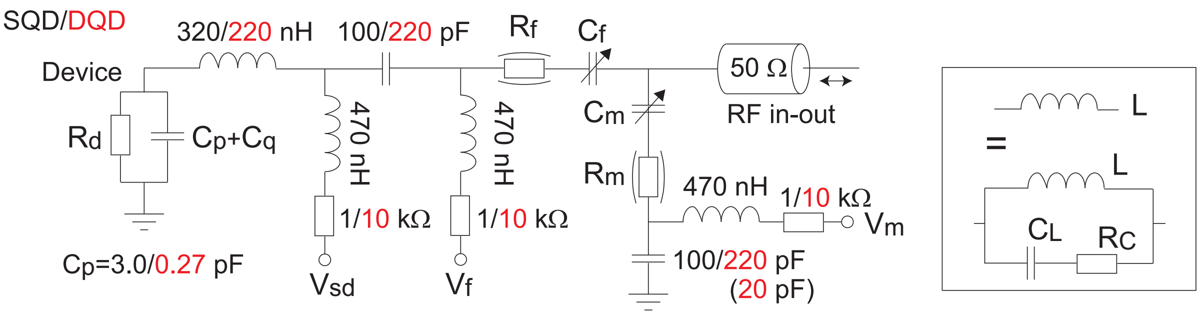

Supplementary Figure 2. Circuit model used to simulate the quantum devices response. The varactors are taken as lumped elements with voltage tunable capacitances C_f, C_m and effective series resistances R_f, R_m, respectively. To take into account the self capacitance of the inductors, these were modelled to be parallel with a capacitance and resistor as indicated by the rightmost panel. The black/red numbers indicate the values of the components used for the single quantum dot (SQD) and double quantum dot (DQD) measurements, respectively.

II ASSEMBLED CIRCUIT

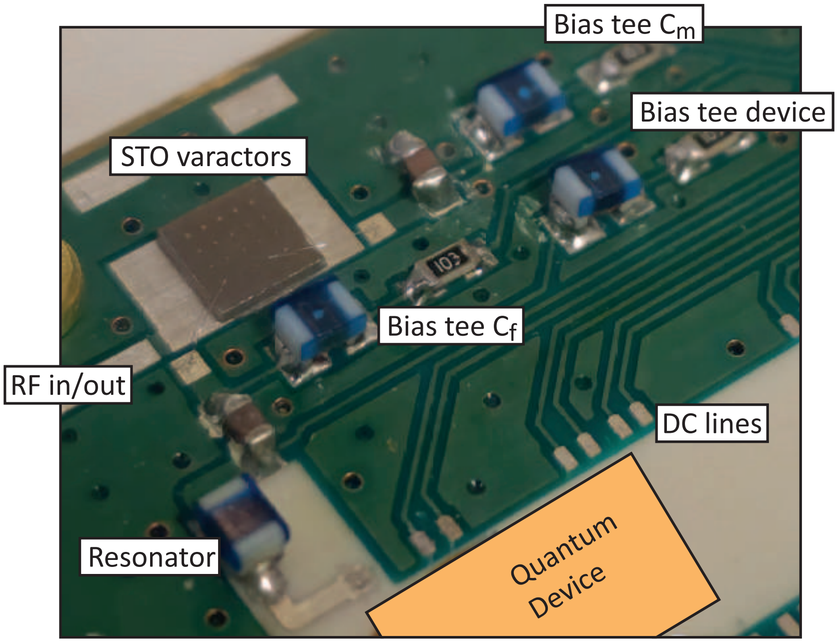

Supplementary Figure 3. Photograph of an assembled circuit, showing the STO varactors as well as the various bias-tees to control the varactors (C_f and C_m) and the quantum device(s). Also labelled are the inductor that forms part of the resonator circuit, the rf in/out line as well as various dc control lines.

III KTO TUNEABILITY

Motivated by the potential of lower dissipated losses [4], as compared to $SrTiO_3$, we also characterised the response of $KTaO_3$ varactors (0.5 mm thickness, single-crystal (100), single-side polished, purchased from SurfaceNet). The fabrication procedure, dimensions, and measurement set-up for the $KTaO_3$ varactors characterisation were identical to that of the $SrTiO_3$ varactors described in Fig. 2 in the main text. The corresponding results for the $KTaO_3$ varactors are summarized in Supplementary Figure 4 below. As is the case for $SrTiO_3$, the $KTaO_3$ varactors are voltage tunable. A capacitance maximum is observed around $V_f = -25$ V and matching conditions are seen around $V_f = 8$ V. As expected, we find that the $KTaO_3$ varactors are less tuneable and have a somewhat smaller overall capacitance as compared to $SrTiO_3$. The observed dissipative losses, however, are not significantly lower than that of the $SrTiO_3$ varactors which is not currently understood. One possibility is that the very low loss tangents previously reported for $KTaO_3$ crystals are typically measured across relatively large (mm size) areas while the varactors used here are of order 100 μm size and edge effects or charge defects might be more important. A systematic study of $KTaO_3$ varactors at mK temperatures of different sizes, geometries and crystal orientations would be of interest.

IV VARACTOR HYSTERISIS AND RESONANCE STABILITY

Strontium titanate shows hysteretic behaviour [5, 6] resulting in a dependence of the varactor capacitance on the voltage sweep history. To illustrate the degree of hysteresis, we plot in Supplementary Figure 5a for each data point the minimum reflection coefficient magnitude measured over a frequency range of 150 to 200 MHz as a function of V_m and V_f, for two different sweep directions, as indicated by the arrows in the top and bottom panels. The measurement in the top panel is taken from the bottom left to the top right corner (V_m=-30 V, V_f=-24 V to V_m=+30 V, V_f=+24 V) and vice-versa for the bottom panel. The dark blue line in each plot corresponds to pairs of (V_f,V_m) where perfect impedance matching is observed - clearly different for the two sweep directions.

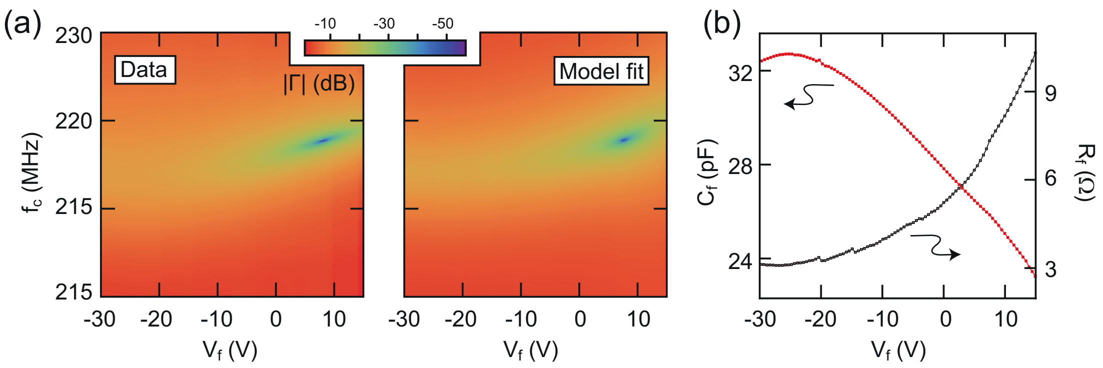

Supplementary Figure 4. **(a)** Colourscale plot of the measured reflection coefficient magnitude $|\Gamma|$ (left) and model fits (right) as a function of rf frequency and varactor voltage V_f for a $KTaO_3$ varactor. For these measurements V_m is set to zero. The data shows shifts of the resonance frequency, reaching a minimum around $V_f \sim -25$ V. The simultaneous change in matching conditions results in a strong variation of the measured magnitude. Perfect matching is observed around $V_f = 8$ V. **(b)** Reflection coefficient magnitude $|\Gamma|$ data and model calculations (solid lines) as a function of frequency for several different varactor voltages as indicated. **(c)** Effective capacitance and resistance values for the varactors obtained from the data fits in panel (a), showing a maximum C_f of 33 pF.

Important for device operation is that after tuning (taking into account the sweep history as illustrated above) the varactors are stable over time and do not add noise to the measurements - a possibility given that the varactors are tunable using electric fields and are, therefore, susceptible to charge noise. To illustrate this, we measure at the resonance frequency ($f_0 = 173.2$ MHz) over a period of one hour with the varactors set to matching conditions, as shown in Supplementary Figure 5b,c, taking data each minute. In the top panels of Supplementary Figure 5c we plot the quadratures (X,Y), using $X + iY = |\Gamma|e^{i\phi}$. The bottom panel of Supplementary Figure 5c shows the scatter observed in the reflection coefficient, that is, $|\delta\Gamma| = \sqrt{(X-\overline{X})^2 + (Y-\overline{Y})^2}$, where $\overline{X}$ and $\overline{Y}$ are the mean values taken over the measurement. No long term drift is observed in $|\delta\Gamma|$.

The measured values for $|\delta\Gamma|$ are consistent with a dominant noise contribution of the low-temperature amplifier (operated at 3 K) in the measurement set-up. This yields an estimate for $|\delta\Gamma|$ of order $\sqrt{P_N/P_C}$, where $P_N = k_B T_N \Delta f$ is the amplifier noise power over a frequency bandwidth Δf, for an amplifier noise temperature T_N, and applied power P_C. For the data in Supplementary Figure 5c we have $T_N \approx 5$ K, a resolution bandwidth $\Delta f = 100$ Hz and applied power $P_C = -95$ dBm, yielding $|\delta\Gamma| \approx 10^{-4}$ consistent with our data. We therefore conclude that the scatter observed in the reflection coefficient is dominated by amplifier noise and other noise sources (including possible noise introduced by the varactors) will have much smaller contributions.

[1] D.M. Pozar, *Microwave Engineering*, 4th ed. (John Wiley & Sons, New York, 2012).
[2] S. Simbierowicz, V. Vesterinen, L. Grönberg, J.S. Lehtinen, M. Prunnila, and J. Hassel, Superconductor Science and Technology. **31**, 105001 (2018).
[3] Zurich Instruments UHFLI, user manual.
[4] R. G. Geyer, B. Riddle, J. Krupka, and L. A. Boatner, Journal of Applied Physics. **97**, 104111 (2005).
[5] A.D. Caviglia, S. Gariglio, N. Reyren, D. Jaccard, T. Schneider, M. Gabay, S. Thiel, G. Hammerl, J. Mannhart, and J.-M. Triscone, Nature **456**, 624 (2008).
[6] D. Davidovikj, N. Manca, H.S.J. van der Zant, A.D. Caviglia, and G.A. Steele, Phys. Rev. B **95**, 214513 (2017).

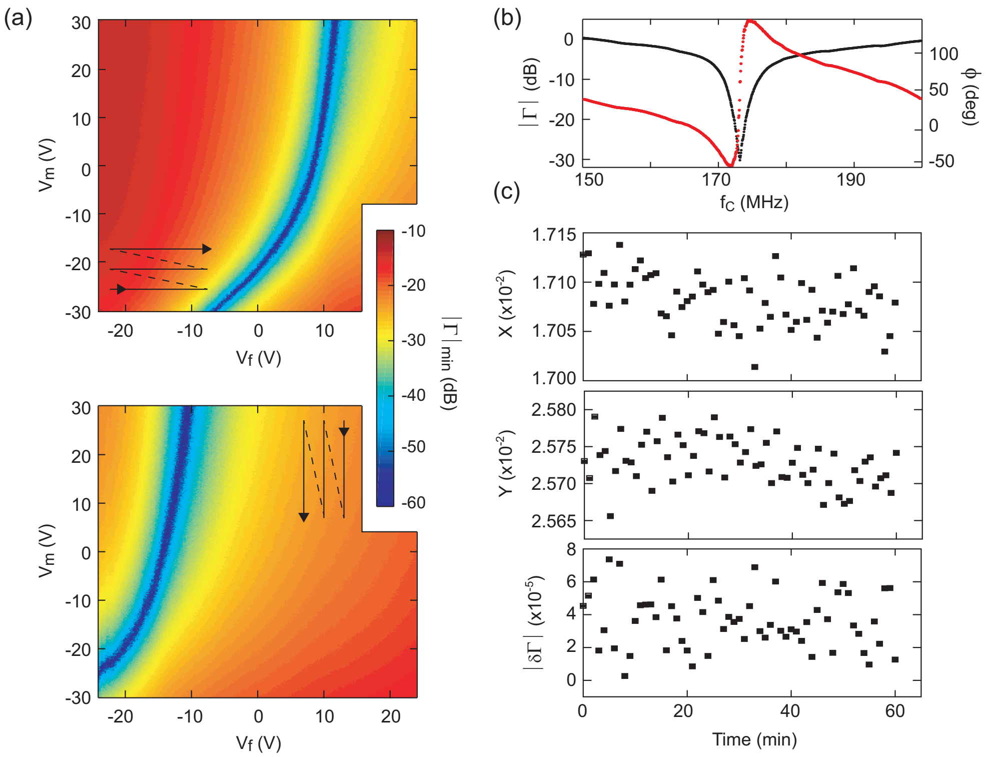

Supplementary Figure 5. **(a)** Colourscale plots showing, for each data point, the minimum reflectance coefficient magnitude $|\Gamma|$ measured over a frequency range of 150 to 200 MHz as a function of V_m and V_f. The top panel shows the result for a sweep direction along V_f while V_m is stepped; from the bottom left to the top right corner (V_m=-30 V, V_f=-24 V to V_m=+30 V, V_f=+24 V) and the opposite sweep and step directions for the bottom panel, as indicated. **(b)** Measured reflection coefficient magnitude and phase response of the device with the varactors set to matching conditions **(c)** Measured quadratures X, Y (top panels) and variation of the reflection coefficient $\delta\Gamma$ over a one hour time period as described in the text.
